# Forming Technologies and Mechanical Properties of Advanced Materials—Second Edition

**DOI:** 10.3390/ma19091778

**Published:** 2026-04-27

**Authors:** Tomasz Trzepieciński, Valentin Ştefan Oleksik, Sherwan Mohammed Najm

**Affiliations:** 1 Department of Manufacturing Processes and Production Engineering, Faculty of Mechanical Engineering and Aeronautics, Rzeszów University of Technology, Al. Powst. Warszawy 8, 35-029 Rzeszów, Poland; 2 Faculty of Engineering, Lucian Blaga University of Sibiu, 550024 Sibiu, Romania; valentin.oleksik@ulbsibiu.ro; 3 Kirkuk Technical Institute, Northern Technical University, Kirkuk 36001, Iraq; sherwan.mohammed@gpk.bme.hu

The technologies for forming metallic and polymeric materials are the most important manufacturing methods used in industry today. The contemporary metal industry is undergoing a dynamic transformation driven by the development of modern technologies. Innovative processes in this industry have increased production efficiency and opened up new opportunities for implementing advanced solutions. Owing to automation and the integration of Industry 4.0/5.0 techniques, the metal industry is becoming an increasingly complex economic entity. Whereas Industry 4.0 focused on implementing automation, artificial intelligence (AI), the Internet of Things, and real-time data analysis, Industry 5.0 is emphasizing human collaboration with technology and the individualization of production. Currently, the implementation of Industry 6.0, defined as virtualized and sustainable manufacturing, has been announced, where the focus is hyperconnected factories and dynamic supply chains [1].

Metals, polymers, and composite materials are widely used in sectors such as the construction, aerospace, and transportation sectors. The introduction of new, innovative materials that are stronger and lighter requires advanced and innovative forming technologies for their production, processing, and joining. Currently, experimental research is being integrated with numerical simulations and generative AI techniques. However, the simulation methods for predicting microstructure, material behavior during deformation, and operation still require engineering expertise to control the product design process. By integrating computational methods with experimental modeling, the time required for experimental testing can be minimized.

Although considerable progress has been achieved in the experimental research, numerical modeling, and optimization of material shaping processes, this progress remains dispersed across research areas. The modern metal industry currently requires approaches from multiple scientific disciplines from metallurgy and plastic forming to computer simulations and artificial intelligence. Therefore, the need arose for this Special Issue to allow advanced researchers to systematically present their studies within a broad thematic context. This Special Issue facilitates interdisciplinary exchange between the academic community and the industrial environment.

This Special Issue showcases the most recent advancements in the forming technologies and mechanical properties of advanced materials. This Special Issue focuses on computational and experimental techniques for joining and metal-forming processes, the microstructure and mechanical properties of advanced materials, tool wear resistance and friction in metal forming operations, and innovative technologies for metal forming. This Special Issue includes 21 original articles and one review by authors from different countries. Specifically, the following research topics are covered in this Special Issue:

The pursuit of rapid prototyping and personalized product manufacturing has led to the intensive development of incremental sheet forming (ISF) methods, specifically single-point incremental forming (SPIF) [2,3]. This method involves gradually deforming sheet metal using a mandrel moving along a preprogrammed trajectory. The main quality limitations of this process include high elastic strain values [4], substantial sheet metal thinning [5], and the low surface quality of the processed product [6] due to the severe frictional interactions between the sheet metal and tool. One method for reducing the friction and plastic resistance of the material is ultrasonic vibration-assisted SPIF. Żaba et al. confirmed the applicability of the SPIF process for forming difficult-to-form Zn-Cu-Ti alloy sheets (Contribution 1). Khalaf et al. investigated the load-bearing capacity of SPIFed EN AW-2024-T3 drawpieces (Contribution 2). The formed stiffening ribs increased the load-bearing capacity of the drawpieces against bending and buckling compared with that of the original material. Rosca et al. examined the potential for the optimal reduction in thickness in the SPIF of polymeric sheets (Contribution 3). These authors determined the optimal conditions for forming polyethylene and polyamide sheets. Lv et al. used ultrasonic-vibration-assisted ISF to form EN AW-1060 aluminum sheets. This innovative forming technology offers benefits in terms of increased surface quality and machining efficiency (Contribution 4). In addition to ISF methods, additive manufacturing (AM) methods, commonly known as 3D printing [7,8,9], involve a similar incremental manufacturing process. The main advantages of AM techniques include lower production costs [10], increased production efficiency [11], and enabling he integrated production of complex structures [12]. These aspects are reflected in the research results presented in this Special Issue. Jabłońska and Łastowska determined the influence of fused deposition modeling (FDM) parameters on the surface roughness of a turned component composed of polyethylene terephthalate glycol (Contribution 5). The researchers confirmed that the additional use of turning, combined with reductions in the feed rate and printed layer height during FDM, reduced the surface roughness of the components. Francese et al. analyzed the dynamic response of 3D-printed acrylonitrile butadiene styrene beams containing metal stiffeners to load, which were developed to add local stiffness to a cantilever beam (Contribution 6). These authors are some of the first to investigate oblique defects starting from bolt holes.

The metalworking industry requires innovative manufacturing methods that are tailored to the increasing strength requirements for products. The behavior of a material during deformation strongly depends on the geometry of the formed products, which directly affects the geometric and surface quality of the part [13]. Achievements in the analysis of plastic working processes, such as rotary swaging [14], hollow sinking [15,16], cold rolling [17], conveyor driver forging [18,19], forming using flexible tools [20], and radial rolling [21,22] are included in this Special Issue. Kocich et al. prepared directly consolidated, nano-sized Y_2_O_3_ with oxide dispersion, which strengthened ferritic steel powder using a rotary swaging process (Contribution 7). Sakaguchi et al. clarified the change in surface roughness that occurs during the hollow sinking of stainless steel SUS304 microtubes (Contribution 8). The authors explained the evolution of surface roughness during hollow sinking. Yang et al. found that the plastic deformation of the Invar36 alloy during cold rolling depended on the grain orientation, resulting in an increase in surface roughness and different slip band morphologies (Contribution 9). Hawryluk et al. developed innovative technology for producing forgings with a double-sided flange (Contribution 10). Balcerzak et al. investigated the deformation potential of Inconel 718 nickel alloy sheets during flexible forming (Contribution 11). Based on the results of finite element-based simulations, the hardness of the elastomeric tool was found to be crucial for ensuring accurate sheet metal processing. Gontarz et al. analyzed the dimensional accuracy of steel rings produced via radial rolling (Contribution 12). They developed guidelines for the process design, considering the influence of feed speed and temperature on the final dimensions of C45 steel forgings. Mróz et al. developed a mathematical model that was combined with finite element-based numerical simulation results to determine the arrangement of straightening rollers in a pre-leveler (Contribution 13). The study material was hot-rolled S235 + JR steel sheet. The removal “coil memory” was removed, reducing the waviness of straightened sheet surfaces.

Manufacturing tools with increasingly improved properties are the foundation of every modern technology. Increasing tool durability justifies the increased cost of the material used [23,24]. In the case of tools used in metal forming processes, increased durability is directly related to friction conditions [25,26]. The appropriate steel grade for tools, heat treatment, wear-resistant coatings, and lubrication conditions adapted to the lubrication conditions and contact pressures [27,28,29] as well as the surface roughness of the friction pair elements [30,31] must be selected. Product surface topography formation and the relationship between process parameters and friction conditions have been analyzed using artificial intelligence methods, especially machine learning (ML) [32] and artificial neural networks (ANNs) [33]. Hawryluk et al. comprehensively investigated the wear of NC11LV steel tools used in extrusion processes (Contribution 14). The authors found that additional hardening through nitriding extended tool life. The latest developments in the durability of forming tools were reviewed by Ficak et al. (Contribution 15). Trzepieciński et al. used ML and ANNs to evaluate the frictional performance of EN AW-5251 sheets (Contribution 16). The sensitivity of 12 activation functions to the accuracy of ANN friction coefficient prediction was examined. Szwajka et al. experimentally tested the effect of cutting parameters on the surface integrity of AISI 316L steel (Contribution 17). The authors found that the feed rate was the most important parameter affecting the surface roughness parameters of the machined surface.

The need for weight reduction in the automotive and automotive industries is driving the increased use of lightweight materials and the development of joining technologies that require no or minimal filler material. Friction stir welding (FSW) is a solid-state welding process that allows for the rapid production of homonymous and dissimilar joints. However, the selection of optimal welding process parameters remains an unresolved issue [34,35]. Another common method for joining metals in the automotive and aerospace industries is adhesive bonding. The research in this area is currently focused on optimizing the welding parameters, surface integrity [36], and surface quality of joined components [37,38,39]. Alkhafaji et al. investigated the effect of tool geometry on the tensile properties of friction stir spot-welded EN AW-6061-T6 aluminum alloy sheets (Contribution 18). They report that a conical pin profile tool composed of AISI H13 steel has improved mechanical properties. Ciecińska et al. analyzed the feasibility of fiber laser surface treatment (FLST) for modifying the surface of EN AW-1050A, EN AW-2024 and EN AW-5083 sheets before the gluing process (Contribution 19). They found that FLTS is a useful technique for increasing the load-bearing capacity of the joint compared with that achieved with sandblasting surface treatment. Podulka et al. used ML methods for selecting the cut-off size and the filtration method for measuring surface roughness (Contribution 20). They demonstrate the potential of the support vector machine technique for optimizing the processes for measuring the surface and analyzing surface topography. Antosz et al. investigated ML algorithms for predicting surface roughness in the milling of 42CrMo4 steel samples (Contribution 21). These authors concluded that the implementation of real-time systems ensures consistent surface quality throughout the milling process.

Due to their high strength, composite materials are widely used, and their number of potential applications is increasing. The main advantage of composite materials is the freedom in selecting materials for the final composite structure [40]. Combining two or more components into a composite material offers almost unlimited possibilities for optimizing physical and mechanical properties. Selecting the matrix and reinforcing phase material is difficult because the choice is affected by the properties of the target product [41]. An analysis of the literature indicates that although the impact of fiber reinforcement on achieving optimal material properties and the associated challenges have been discussed in detail, most studies have not considered the combined effects of key fiber parameters, such as fiber shape and type [42,43]. Khalel and Khan proposed a model for predicting the compressive and flexural strengths of fiber-reinforced concrete (Contribution 22). Based on the proposed model, an optimal set of fiber properties can be selected during reinforcement.

In conclusion, the second edition of this Special Issue focuses on the most recent advances in various technologies for forming materials and the mechanical properties of these materials, emphasizing the role of advanced forming routes and comprehensive characterization in the analysis and testing of high-performance materials. The contributions included in this Special Issue strengthen the link between the properties of advanced materials, their processing methods, and their industrial applications. The guest editors hope that the articles in this Special Issue will provide valuable resources for industry professionals and researchers, promoting the use of more environmentally friendly materials and technologies in sustainable industries.

In the future, further research on the synergistic effects of the microstructure, properties, and applications of materials will provide the basis for transforming manufacturing methods toward a sustainable industry. In the era of Industry 4.0, metal forming methods are evolving, incorporating AI techniques and numerical simulations into product design and manufacturing. Advanced high-strength materials enable reductions in component weight and increases in fatigue resistance. Additionally, aspects of Industry 4.0 has raised concerns about the ethical use of AI and AI-induced human unemployment. The goal of the transition from Industry 4.0 to Industry 5.0 is to restore human involvement in manufacturing processes, considering sustainable development and efficient, performance-oriented production. A collaborative approach between machines and humans, integrating cobots and digital twins, is key to a sustainable future for the manufacturing industry and the use of environmentally friendly materials.
